# Cognitive Remediation and Social Recovery in Early Psychosis (CReSt-R): protocol for a pilot randomised controlled study

**DOI:** 10.1186/s40814-022-01064-6

**Published:** 2022-05-24

**Authors:** E. Frawley, M. Cowman, M. Cella, D. Cohen, E. Ryan, B. Hallahan, C. Bowie, C. McDonald, D. Fowler, T. Wykes, G. Donohoe

**Affiliations:** 1 Centre for Neuroimaging, Cognition & Genomics (NICOG), School of Psychology, National University of Ireland, Galway, Ireland; 2 Institute of Psychiatry, Psychology & Neuroscience, King’s College, London, England; 3 South Galway Child & Adolescent Mental Health Service, Health Service Executive, Merlin Park Hospital, Galway, Ireland; 4 Department of Psychiatry, National University of Ireland, Galway, Ireland; 5 Psychology Service, Adult Mental Health Service, University Hospital Galway, Galway, Ireland; 6 Department of Psychology, Queen’s University, Kingston, ON Canada; 7 Department of Psychology, University of Sussex, Brighton, England

**Keywords:** Early psychosis, Psychosocial intervention, Social function, Occupational function, Social recovery, Cognitive remediation, Pilot, Feasibility

## Abstract

**Background:**

Psychosis, even in its early stages, is associated with significant disability, causing it to be ranked ahead of paraplegia and blindness in those aged 18–35 in terms of years lived with disability. Current pharmacological and psychological interventions intervention have focused primarily on the reduction of positive symptoms (hallucinations and delusions), with little benefit to domains of psychosis such as cognitive difficulties and social and occupational functioning.

**Methods/design:**

The CReSt-R intervention trial is a single center, pilot randomised controlled study based at the National University of Ireland (NUI), Galway. The trial will recruit participants from four clinical sites with assessment and intervention completed by the primary NUI Galway team. The trial will explore the feasibility, acceptability, and effectiveness of a novel psychosocial intervention for early psychosis based on a combined cognitive remediation training and cognitive behavioural therapy approach focused on social recovery. Participants, aged 16–35 within the first 5 years of a diagnosed psychotic disorder, will be recruited from the Children and Adolescent Mental Health Service and the Adult Mental Health Services in the region.

**Discussion:**

Cognitive remediation training (for improving cognition) and social recovery focused cognitive behavioural therapy, have both separately demonstrated effectiveness. This trial will evaluate the feasibility, acceptability, and explore the efficacy of a treatment approach that combines both approaches as part of an integrated, multicomponent intervention.

**Trial registration:**

Cognitive Remediation & Social Recovery in Early Psychosis (CReSt-R): ClincialTrials.gov Identifier NCT04273685. Trial registered Feb 18^th^, 2020. Last updated April 14^th^, 2021.

## Background

In psychosis spectrum disorders, there has been a shift in focus from research and development focused purely on pharmacological symptom management to a focus on the broader concept of recovery. Although anti-psychotic medications have been effective in symptom remission, less than half of all schizophrenia patients have been able to achieve recovery [1]. Residual impairments in both neurocognition and social cognition, unaddressed by pharmacological intervention, continue to have a significant impact on function and the rate of disability in those living with psychosis [2, 3]. The rate of development of new pharmacological interventions has slowed with no new drug released to the market in approximately 20 years.

While cognitive deficits and their impact on the social and occupational functioning are well established in chronic schizophrenia, their effects in early psychosis (defined as within the first 5 years of a diagnosed psychotic disorder) are less well understood. A meta-analysis recently published by our group explored cognitive predictors of social recovery in early psychosis using cross-sectional and longitudinal data. The meta-analysis comprised 46 studies including 3767 participants and was based on nine cognitive domains. All cognitive domains were related to psychosocial function both cross-sectionally and longitudinally. These associations remained significant even after the effects of symptom severity, duration of untreated psychosis (DUP) and length of illness were accounted for. General cognitive ability (IQ) and social cognition were most strongly associated with both concurrent and long-term function [4].

To understand the relationship between *remission* and recovery in early psychosis, remission has been defined as referring to symptomatic and/or functional improvement over a > 6-month time frame and using specific assessment criteria (The Remission in Schizophrenia Working Group RSWG criteria). *Recovery* on the other hand was defined as symptomatic and functional improvement in social, occupational, and educational domains over a time frame of > 2 years [5]. In their meta-analysis of long-term outcome studies of first episode psychosis (FEP) 58% of participants met remission criteria at a mean of 5 years and 38% met recovery criteria at a mean of 7.2 years.

Key elements of recovery from an individual perspective have been identified as including connectedness, hope, identity, empowerment, and having a meaningful role [6]. However, these concepts are difficult to operationalise and quantify at a service level and so may get ‘lost in translation’ using conventional outcome measures, such as hospital admission rates, symptom reduction or global level of functioning.

In a systematic review and meta-analysis of 31 studies, we [7] investigated the impact of current psychosocial intervention on social and occupational functioning (both global and individual). We found that cognitive remediation training (CRT) was associated with significant gains in function, similar to chronic schizophrenia. CRT is defined as ‘a behavioural training-based intervention that aims to improve cognitive processes [attention, memory, executive function, social cognition, or metacognition] with the goal of durability and generalisability’ (‘Cognitive Remediation Experts Workshop (CREW)’, Florence, April 2010).

Cognitive Behavioural Therapy for psychosis (CBTp) is an evidence-based talking therapy with the primary aim of reducing clinical symptom severity, e.g. hallucinations and reducing relapse rates. This type of therapy was not significantly associated with improved social and occupational functioning. However, CBT focused on social recovery, social recovery therapy (SRT), was associated with significant improvements. Multicomponent interventions were found to be associated with the strongest gains in social and occupational functioning [7]. Across psychosis spectrum disorders, social cognition has been repeatedly linked to functional outcomes [8–11]. Social cognition is reported to mediate the effects of neurocognition on functional outcomes [2, 12–14].

Early intervention in psychosis (EIP) services are multi-disciplinary, clinical teams established to seek, identify, and reduce treatment delays at the onset of psychosis. They promote recovery by providing evidence-based intervention thereby reducing the probability of relapse following a first episode of psychosis. The concept, purpose, and effectiveness of *multicomponent intervention* in EIP has been described previously [15]. These interventions included the ‘core’ components of psychopharmacological treatment (with regular medication review) and family psychoeducation/counselling, alongside ‘optional’ components of CBT, family therapy, vocational and education counselling, social skills training, crisis management, and a crisis response team. Where does cognition fit in this multicomponent model?

Previously, in a review of social cognitive interventions, it was concluded that in order to impact higher-order social cognitive processes, there needs to be ample opportunity for practice of skills both in a clinical setting as well as in the community [16]. Social cognition is reported to mediate the effects of neurocognition on functional outcomes [2, 12, 14]. This suggests better functional outcomes may be achieved if both neurocognition and social cognition are targeted in intervention and that neurocognitive training alone does not result in significant social cognitive improvements [3, 14].

The CReSt-R study investigates a novel approach to optimising the cognitive and functional benefits of psychological interventions in early psychosis. It involves a multicomponent intervention that combines (a) CRT- a Computerised Interactive Remediation of Cognition-Training for Schizophrenia (CIRCuiTS) [17–19] with (b) social recovery therapy (SRT) [20–22]. In so doing, the aim is to target both social and occupational functioning and social cognition in young people living with psychosis, two outcomes of interest for this study.

CRT is recognised as an effective treatment in schizophrenia generally with a large meta-analysis reporting an effect size of Cohen’s *d* = 0.45 for cognitive performance, *d* = 0.42 for psychosocial functioning and *d* = 0.18 for symptom severity [23]. CRT programmes have evolved over the years with a variety of programme protocols and specific techniques now reported in the literature. An expert working group, identified four core features of CRT, including facilitation by a therapist, cognitive exercise, procedures to develop problem-solving strategies, and procedures to facilitate transfer to real-world functioning [24]. A meta-analysis supports this emphasis, finding that better outcomes following CRT were associated with an active and trained therapist, structured development of cognitive strategies, and integration with psychosocial rehabilitation [25]. The CIRCuiTS programme, outlined in the “Methods/design” section below, embodies these core elements. It is also informed by a metacognitive model, emphasising self-awareness, self-monitoring, and self-direction when completing the programme tasks and the transfer of these skills to everyday life.

SRT is informed by cognitive behavioural theory. It is an evolved form of cognitive behavioural therapy (CBT) with an emphasis on assertive outreach and behavioural experimentation. Similar to the CIRCuiTS programme it aims to apply cognitive work and newly acquired knowledge and strategies to everyday life with a focus on self-awareness and self-monitoring.

The CReSt-R study will contribute to the cognitive remediation field and the wider field of recovery in early psychosis by exploring the feasibility, acceptability, and effectiveness of this multicomponent psychosocial intervention with the hypothesis of a greater impact on social and occupational functioning and social cognition compared to treatment as usual in the target group. Whilst both intervention components have demonstrated efficacy in previous studies in addition to being found acceptable to participants [17–22], the acceptability of the combined, multicomponent intervention to young people aged 16–35 in the early psychosis population is unknown. In addition, the feasibility of delivering the multicomponent intervention and running a larger scale randomised control trial in Ireland is unknown.

## Methods/design

### Aims and objectives of the CReSt-R pilot randomised controlled study

The aim of the CReSt-R pilot randomised control study is to gather and analyse acceptability and feasibility data to (1) further develop and refine the novel, multicomponent CReSt-R intervention (2) investigate the feasibility of delivering and evaluating the intervention in future definitive trials. Specifically, the study objectives (outlined in further detail in “The CreSt-R intervention and control condition”, “Feasibility”, “Acceptability”, “Estimating treatment effect sizes” sections) include the following:

1. To collect qualitative and quantitative data to assess the feasibility of the intervention with indicators in the areas of process, intervention, and resources.
2. To investigate if the CReSt-R intervention is acceptable to young people, aged 16–35, who are within the first 5 years of a diagnosed psychotic disorder.
3. To explore the effectiveness of the intervention by analyzing primary and secondary outcome data to provide treatment effect estimates, thus informing future trial design.

### Ethics, consent, and permissions

This study was approved by the Galway Clinical Research Ethics Committee, Merlin Park Hospital, Galway, Ireland. All participants will provide informed signed consent. The ethics application also detailed general data protection regulation (GDPR) considerations, the proposed management of vulnerable individuals in the study and assent for participants aged under 18 years of age.

### Setting and participants

This is a community-based study and will recruit participants from the Children and Adolescent Mental Health Service (CAMHS) and the Adult Mental Health Service (AMHS). Recruitment referrals from primary care providers and self-referrals are also accepted on a case-by-case basis with a primary clinical contact deemed essential for participation. Collaboration with clinical teams is anticipated to assist with recruiting adequate number of participants for this study. A sample size of 30 is a common ‘rule of thumb’ in pilot studies [26, 27], with 15 in the intervention arm and 15 in the control arm considered adequate in generating data to explore the feasibility and acceptability of the proposed intervention and in providing an estimate of the intervention’s efficacy for planning a definitive intervention trial. This pragmatic approach is consistent with other feasibility studies in the area of early psychosis [28] and in line with current recommendations for pilot studies [29].

Inclusion criteria for the study are being aged between 16 and 35 years of age, within the first 5 years of a diagnosed psychotic illness (based on time since first contact with a clinical service), community based, clinically stable and having the ability to give consent. Exclusion criteria are having a history of organic impairment (including IQ < 70), history of a head injury with loss of consciousness > 5-min duration and drug abuse in the preceding month. Confirmation of diagnosis, timeframe of onset of psychotic symptoms, presence of cognitive and social and occupational difficulties will be provided via a referral form completed by the primary clinical contact. Participants may withdraw from the study at any time.

### Study design, randomisation, and treatment allocation

**Fig. 1:**
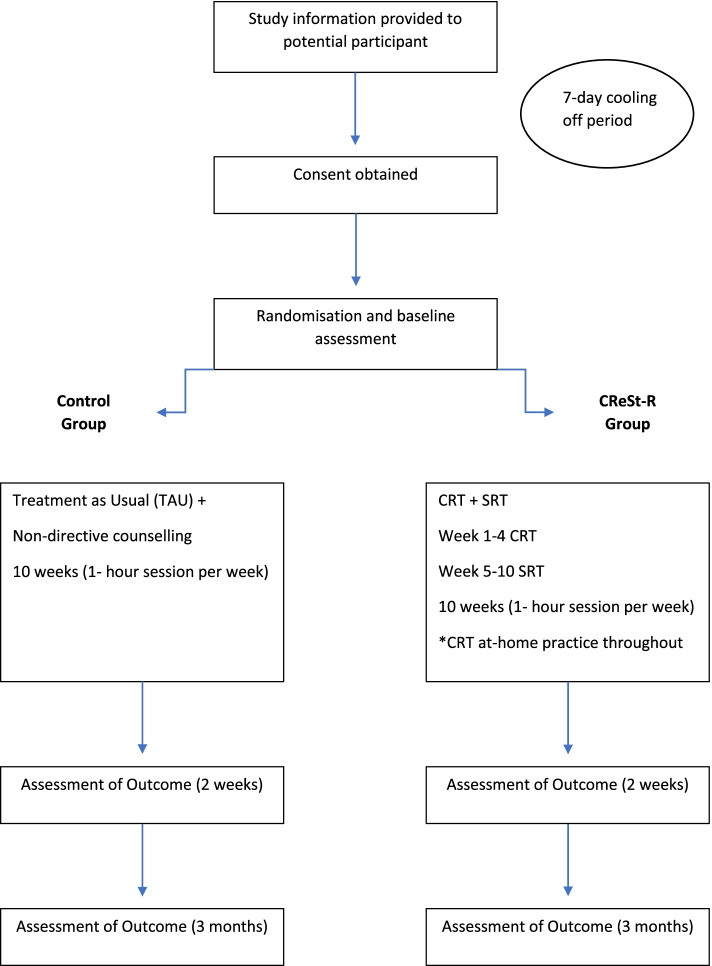
CReSt-R Consort Diagram

A randomised pilot study with a controlled, outcome-assessor-blind, parallel- group design will be implemented. Randomisation will use a permuted block design, using a computerised random number generator with predetermined 1:1 allocation ratio and will be completed by an independent statistician. The study research assistant will provide an information sheet to a potential participant and answer any questions they may have before obtaining written consent. There will be a 7-day cooling off period between provision of consent and enrolment to the study. Upon enrolment into the study the participants will be randomised to the intervention group (CReSt-R) or the control group. Both interventions are detailed below. After randomisation, the participant will complete baseline assessments with an assessor blind to treatment allocation. All participants will be instructed not to reveal their treatment allocation prior to each follow-up assessment. Should the blind be broken for any participant, this will be noted and reported to the principal investigator. The primary clinical contact for each individual participant will be informed of treatment allocation. The consort diagram for study procedure is contained in Fig. 1.

### The CreSt-R intervention and control condition

#### Component 1

The CRT programme used in this study is the Computerised Interactive Remediation of Cognition-Training for Schizophrenia (CIRCuiTS). CIRCuiTS is a web-based CRT programme which targets metacognition, specifically strategy use, in addition to massed practice of cognitive functions (attention, memory, and executive functioning). Collaborative goal setting related to real-world tasks are integral to the programme with the programme tasks and exercises increasing in difficulty in response to the participant’s performance and progress. The protocol for CIRCuiTS training will follow that of a previous efficacy study [19]. This will be the primary focus of 1:1 therapy for the first weeks with remote practice sessions occurring between therapy visits. After 4 weeks, remote practice will continue and the focus of in-person therapy sessions will bridge to Social Recovery Therapy as detailed below.

#### Component 2

Social recovery therapy (SRT) focuses on addressing barriers to individuals interacting in their social environment, e.g. social anxiety. It is informed by cognitive behavioural theory and addresses individual goals. SRT follows an established protocol [20, 21]. In summary, this consists of therapy delivered in three stages. Stage 1 will include engagement and formulation with the purpose of identifying a problem list and establishing a therapeutic relationship. Stage 2 will include preparing for new activities with identification of pathways to activity and collaboration with community stakeholders. Stage 3 will include engagement in new activities using behavioural experiments to promote social activity. This is the primary focus of in-person therapy sessions from week 5 to 10 alongside remote practice of the CRT programme. There is emerging evidence to support brief intervention in both CRT [30] and CBTp [31]. Rationale for intervention duration in the CReSt-R study builds upon this recent work in addition to a previous study by our group which reported significant gains in both neuropsychological function and social function at follow-up post an 8-week, low support, remotely accessible CRT programme for chronic psychosis [32]. Intervention duration will also be considered as a feasibility indicator in this study.

In the control group of the study participants will receive treatment as usual (TAU) plus 10 weeks of 1:1 non-directive counselling matching the intervention group for time. This consists of 10 1:1, hour-long sessions with the same intervention therapist who delivers the CRT intervention. The therapy in the control condition is characterised by empathy, unconditional positive regard, congruence, and non-directivity. Notes pertaining to each session are recorded and clinical supervision is provided by the principal investigator.

The CReSt-R intervention was initially intended for delivery in in-person sessions with a strong emphasis on assertive outreach, community-embedded intervention delivery, and therapeutic rapport. However, in response to the COVID-19 pandemic and resulting public health guidelines, the protocol was revised to enable adaptation to these circumstances. The outcome measures and delivery of the intervention can now be offered face to face, entirely online, or in a blended approach remaining true to the core therapeutic principles of both components of the multicomponent intervention. These changes reflect broader change in clinical practice in response to the global pandemic and identified opportunities in this area of intervention delivery [33]. The delivery mode of the intervention will be considered in the analysis and interpretation of results.

### Feasibility

All statistical analyses will occur after completion of data collection and will adopt the intention-to-treat (ITT) principle. All data will be processed in SPSS version 27. The first objective of the analysis, assessing feasibility, will consist of descriptive statistics with derivation of means and standard deviations or medians, minimum/maximum values and interquartile range for continuous measures and proportions for ordinal or multinomial categorical and binary coded measures as appropriate. Participants’ baseline demographics and clinical characteristics will also be reported. Missing data patterns will be described for all three outcome time points.

*Process* feasibility indicators include recruitment and retention rates reported per month of trial and in total at trial completion, appropriateness of inclusion criteria and reasons for exclusion from the trial as reported by clinical collaborators, effectiveness of randomisation procedure, and effectiveness of blinding procedures.

*Intervention* feasibility indicators include participant adherence to the trial protocol, intervention duration/therapy dosage and therapy fidelity.

*Resource* feasibility indicators include therapist time in session, remote support, and clinical supervision; intervention costs for software, running costs, and participant reimbursement for assessment sessions. See Table 1 for further detail of assessment of feasibility indicators.

**Table 1:** Feasibility indicators assessment

Feasibility indicator	Assessment
^a^Recruitment rate	% of participants recruited/time
^b^Retention rate	% of participants who complete T1, T2, and T3 outcome assessments Descriptive data on participants who leave the study early-therapy group (intervention v’s control), # of sessions completed, cited reason for leaving.
Inclusion criteria	Completion rate of referral form by clinical contact Descriptive data on reasons for exclusion from study % of participants referred to study who meet inclusion criteria
Randomisation procedure	Evaluation of 1:1 ratio at end of trial (# of intervention participants: # control participants) Logged data on any errors made
Blinding procedure	Blinding in this trial will be assessed by asking blinded assessors to guess the trial group assignment and comparing these responses to what would be expected by chance Logged data on unblinding occurrences during trial
Adherence/intervention duration/therapy dosage	# of therapy sessions completed per participant Time spent on CIRCuiTS (at-home work) per participant. (logged on CIRCuiTS software platform) Time spent on at-home behavioural experiments (logged per participant throughout trial)
Therapy fidelity	Completion rate of clinical supervision sessions Completion rate of fidelity checklists
Therapist time- in session	Total time spent by therapist in session and documentation per month (data logged throughout study)
Therapist time- remote support	Total time spent communicating via email, text, or phone outside of therapy session per month (data logged throughout study)
Clinical supervision	# of clinical supervision sessions per month
Software	Total cost of CIRCuiTs license software per month
Running costs	Total cost of study expenses per month, e.g. study phone
Participant reimbursement	Total cost of participant reimbursement for assessment sessions per month
^c^Qualitative study	Reflexive thematic analysis of semi-structured interview data
Intrinsic Motivation Inventory [34]	Completion rate and results of IMI

^a, b, c^ Key feasibility indicators for progression

Criteria for progression to a larger study will be assessed using three key feasibility indicators namely (1) recruitment rate (2) retention rate and (3) acceptability of the intervention. A system of proceed, amend, or stop will be utilised modeled on previously used traffic light systems [35] (see Table 2). This system operates on the use of guidelines rather than strict thresholds in line with current recommendations [35, 37–39]. A decision to progress the trial will be decided by the above criteria, as well as discussion with the study research team, clinical collaborators, and patient–public involvement panel.

**Table 2:** Progression criteria

Key indicator	Proceed	Amend	Stop
**Recruitment rate** *Target figure*: 30 participants	≥ 70% of target number	51–69 % of target number	≤ 50% of target number
**Retention rate** *Target figure*: 75% of participants randomised to intervention group will complete outcome measures at T1, T2, and T3 [36]	≥ 70% of target number	51–69% of target number	≤ 50% of target number
Acceptability	Intervention is described as acceptable by participants in its current form	Intervention is described as acceptable with recommended changes to improve participant experience	Intervention is described as unacceptable by participants
Action	Continue with intervention and study design with collaboration between research team, clinical collaborators, and PPI contributors	Consultation with research team, clinical collaborators, and PPI contributors regarding necessary amendments to the intervention and study design	No progression to further trial

### Acceptability

Acceptability of the intervention will be assessed using the Intrinsic Motivation Inventory (IMI) administered on completion of the study [34]. A qualitative semi-structured interview schedule has also been developed for completion at the end of the intervention (see Appendix 1). This embedded qualitative study will allow participants to provide feedback focusing on the following: their general experience of participating in the intervention, intervention components, experience of recruitment, communication, and perceived benefits and challenges of participating in the intervention. The qualitative data will be analysed using a reflexive thematic analysis approach [40]. The acceptability aspect of this study will be integral in further developing the multicomponent intervention and optimising clinical utility. The interview schedule itself will be reviewed for adaptation for future use based on interviewer and interviewee feedback.

### Estimating treatment effect sizes

To clarify, this study does not aim to determine treatment effect. However, to inform statistical power calculations for primary and secondary treatment outcomes in advance of a full RCT, estimates of treatment effect sizes will be obtained using linear mixed models. These analyses, completed in SPSS version 27, will provide a treatment effect estimate on each outcome measure at 2 and 12 weeks post-intervention. Outcome measures at these two time points will be entered into the model as the dependent variables with fixed effects of study arm, baseline outcome measures, time, and a time point by study arm interaction. Inclusion of baseline outcome measures accounts for their potential prediction of future outcome and will contribute towards accurate effect estimates. A random effect for participant will also be entered into the model to account for correlations between the two time points (repeated measures) per participant. This analysis will be carried out by the trial statistician.

### Assessment battery

#### Primary outcome measure

Social and occupational functioning will be assessed using the Social and Occupational Functional Assessment Scale (SOFAS) [41] with an additional secondary outcome included below.

#### Secondary outcome measures

1. A secondary *social and occupational functioning* measure will be the Time Use Survey [42].
2. *Social cognition* will be measured using a battery of assessments based on the recommendations from the Social Cognition Psychometric Evaluation Study (SCOPE) final Validation Study [11]. These will include (a) The Emotion Recognition Task (ERT) from the Cambridge Neuropsychological Test Automated Battery (Cambridge Cognition Ltd.), (b) the Hinting Task [43], (c) The Bell Lysaker Emotion Recognition Task (BLERT) [44], and (d) the Reading the Mind in the Eyes Task [45] as operationalised in our previous CRT trial [32].
3. *Cognitive function* will be assessed in terms of general cognitive ability, memory function and executive function. General cognitive ability will be measured using the similarities and matrix reasoning subtests from the Wechsler abbreviated scale of intelligence [46]. Memory function will be assessed using the logical memory subtest and the letter number sequencing task from the Wechsler Memory scale 3rd edition [47]. Visual memory will be measured using the Rey Osterreith Complex Figure (ROCF) [48]. Executive functioning will be measured by the STROOP [49].
4. The Intrinsic Motivation Inventory for Schizophrenia Research [34] will assess intrinsic motivation and self-regulation. Subscales of the assessment will include interest/enjoyment, perceived competence, effort, value/usefulness, felt pressure and tension, and perceived choice while participating in the study.
5. The Need for Cognition Scale (NCS) [50] will assess the degree to which participants seek out cognitively challenging activities of daily living and will provide supplementary information to the social and occupational functioning outcome measures.
6. *Clinical Assessment* will include the Positive and Negative Syndrome Scale (PANSS) [51] (see Table 3).

**Table 3:** CReSt-R outcome measures

Primary outcome measure	Social and Occupational Functioning: Social and Occupational Functional Assessment Scale (SOFAS) [41]
Secondary outcome measures	Function: The Time Use Survey [42] Social Cognition: CANTAB Emotion Recognition Task (ERT) The Reading the Mind in the Eyes Test [45] The Hinting Task [43] The Bell Lysaker Emotion Recognition Task (BLERT) [44] General Cognition: Wechsler Adult Scale of Intelligence 3^rd^ edition (WAIS-III)-The similarities and matrix reasoning subtests [46] Wechsler Memory scale 3rd edition- logical memory subtest [47] Rey Osterreith Complex Figure (ROCF) [48] The Stroop Test [49] Clinical Measures: Positive and Negative Syndrome Scale (PANSS) [51] Self-report measures: The Need for Cognition Scale (NCS) [50] Intrinsic Motivation Inventory (IMI) [34]

## Discussion

A strength of the protocol is the novelty of the combined intervention and in particular in the early psychosis cohort. The robust outcome assessment battery will enable us to estimate efficacy parameters for the intervention so as to inform further definitive trials in terms of social and occupational functioning, social cognition, general cognition, and other self-report measures. Data on feasibility key indicators of intervention delivery will also assist us in exploring the potential use of the intervention in clinical practice. Potential limitations of the study include the challenge of recruitment of participants in this difficult to ascertain cohort. It is also noted the varying modes of delivery of the intervention (online, blended, in-person), whilst potentially acceptable to participants, need to be considered as part of the interpretation of data collected in the study and the potential both to inform a definitive trial and/or translate the intervention into clinical practice settings.

### Trial status

This trial is ongoing. Trial Registration: ClinicalTrials.gov Identifier: NCT04273685. First received: February 18th 2020

## Data Availability

The full protocol in addition to datasets and statistical code generated during the current study will be available from the corresponding author on reasonable request.

## Abbreviations

AMHS: Adult Mental Health Service
BLERT: The Bell Lysaker Emotion Recognition Task
CAMHS: Children and Adolescent Mental Health Service
CBT: Cognitive behavioural therapy
CBTp: Cognitive behavioural therapy for psychosis
CIRCuiTS: Computerised Interactive Remediation of Cognition-Training for Schizophrenia
CReSt-R: Cognitive Remediation and Social Recovery in Early Psychosis Study
CREW: Cognitive Remediation Experts Workshop
CRT: Cognitive remediation training
DUP: Duration of untreated psychosis
EIP: Early intervention in psychosis
ERT: Emotion recognition task
FEP: First episode psychosis
GDPR: General data protection regulation
IMI: Intrinsic Motivation Inventory
ITT: Intention to treat
IQ: Intelligence quotient
NCS: Need for cognition scale
NUI: National University of Ireland
PANSS: Positive and Negative Symptom Scale
ROCF: Rey Osterrith complex figure
RSWG: Remission in schizophrenia working group
SCOPE: Social cognition psychometric evaluation study
SMD: Standardised mean difference
SOFAS: Social and occupational functioning scale
SRT: Social recovery therapy
TAU: Treatment as usual
